# Advances in DNAzyme Selection, Molecular Engineering and Biomedical Applications

**DOI:** 10.3390/ijms27041833

**Published:** 2026-02-14

**Authors:** Li Yan, Jingjing Tian, Hongyu Yang, Shuai Liu, Zaihui Du, Chen Li, Hongtao Tian

**Affiliations:** 1 College of Food Science and Technology, Hebei Agricultural University, Baoding 071000, China; 2 State Key Lab of Meat Quality Control and Cultured Meat Development, College of Food Science and Technology, Nanjing Agricultural University, Nanjing 211800, China; tianjingjing@njau.edu.cn; 3 Institute of Food and Nutrition Development, Ministry of Agriculture and Rural Affairs, Beijing 100081, China; 4 Hebei Technology Innovation Center of Probiotic Functional Dairy Product, Baoding 071000, China; 5 National Engineering Research Center for Agriculture in Northern Mountainous Areas, Baoding 071000, China

**Keywords:** DNAzyme, in vitro selection, SELEX, chemical modification, XNA, nanocarriers, biosensing, molecular imaging, nucleic acid therapeutics

## Abstract

DNAzymes are catalytically active single-stranded DNAs that fold into metal-ion-assisted architectures to mediate diverse reactions. Addressing the performance gap in biological settings, we establish a novel conceptual framework based on a continuous iteration workflow of selection, enhancement, and application. This paradigm integrates selection constraints, molecular engineering, and clinical context into a unified cycle. We summarize the evolution of SELEX toward application-driven selection incorporating functional/environmental constraints, deep-sequencing-enabled high-throughput activity readouts, droplet compartmentalization and structure- and computation-guided design. We further consolidate engineering strategies to improve stability, kinetics and controllability, including 2′-sugar modifications and XNA substitution, backbone and nucleobase functionalization, arm and secondary-structure engineering for switchable or split architectures and multivalent organization on nanocarriers or nucleic acid scaffolds to enhance local concentration, protection and targeted delivery. Finally, we survey applications in ultrasensitive biosensing and portable diagnostics, activatable and multimodal in vivo imaging, and therapies for cancer, inflammatory diseases and airway disorders, and outline translational priorities: data-driven design, next-generation delivery, standardized safety/PK-PD evaluation and scalable manufacturing, ultimately for clinical and point-of-care deployment.

## 1. Introduction

DNAzymes are artificially synthesized single-stranded DNA molecules obtained through in vitro selection. By folding into defined and often metal-ion-assisted three-dimensional structures, they form enzyme-like active centers capable of catalyzing diverse chemical reactions. Their catalytic scope extends beyond nucleic acid cleavage to include ligation, chemical modification and other organic transformations [1,2,3,4,5,6,7,8]. Compared with other nucleic acid therapeutic modalities (e.g., siRNAs, antisense oligonucleotides (ASOs), and CRISPR-based systems), DNAzymes are typically short, fully synthetic oligonucleotides that are readily amenable to scalable chemical synthesis and site-specific modification, and they feature a modular architecture in which the catalytic core and target-recognition arms are functionally separable. To avoid overgeneralization, we summarize the positioning of DNAzymes relative to these modalities across mechanisms, dosing implications, off-target liabilities, manufacturing, delivery constraints, and clinical maturity in Table 1. This modularity enables rapid retargeting without altering the catalytic scaffold, conferring attractive potential across disease models as well as in bioimaging, signal amplification and integrated theranostic platforms [9,10,11,12,13,14,15].

Nevertheless, translating catalytic nucleic acids from test tubes to complex biological environments presents challenges. Canonical DNAzymes selected under conventional conditions (e.g., high divalent metal concentrations) may show reduced activity in vivo. However, it is important to note that the inherent adaptability of SELEX allows for the optimization of selection pressures to bridge this gap [29]. Beyond the canonical RNA-cleaving motifs, many DNAzymes have been demonstrated to function in complex biological environments, including live-cell sensing and imaging of physiologically abundant ions (e.g., Na^+^ and Li^+^) as well as redox-active metals (e.g., Fe^2+^/Fe^3+^) [30,31,32,33,34]. Moreover, DNAzyme catalysis is not restricted to phosphodiester cleavage; representative examples include peroxidase-mimicking G-quadruplex/hemin DNAzymes and other chemistries such as photochemical lesion repair and RNA ligation [35,36,37]. DNAzyme-based platforms have also been adapted to recognize and report on higher-order targets, including pathogenic bacteria, enabling whole-cell or pathogen-associated detection schemes [38,39]. In the absence of a detailed understanding of DNAzyme–substrate complex structures and reaction coordinates, stabilization chemistry, cofactor tuning and nanocarrier construction frequently rely on empirical optimization or large-scale trial and error, making it difficult to achieve an optimal balance among catalytic activity, biostability and safety [2,40,41,42,43]. For instance, heavy chemical modifications introduced to enhance nuclease resistance often perturb the native folding of the catalytic core or metal-binding affinity, leading to compromised kinetics [9,44,45]. Conversely, prioritizing high catalytic turnover without sufficient protection results in rapid degradation in physiological fluids, while excessive cofactor requirements may raise cytotoxicity concerns [9,46,47]. To maximize the biomedical potential of DNAzymes, their design and deployment should be re-evaluated and optimized across selection conditions, molecular architecture, and the operating environment.

Over the past decade, multiple technological advances have created new opportunities for DNAzyme redesign and performance enhancement. On the one hand, a range of improved SELEX strategies has emerged, including function-oriented selection in cells or in vivo-like environments, enrichment profiling enabled by high-throughput sequencing and machine learning, and high-throughput single-entity selection based on microfluidics and droplet technologies, collectively making it increasingly feasible to obtain high-performance DNAzymes under physiologically relevant conditions [29,48,49]. On the other hand, rapid progress in nucleic acid chemical modification, DNA nanostructures and multifunctional nanocarriers has enabled fine control of DNAzyme conformation and physicochemical properties at the levels of sugars, bases and backbones, as well as local high-density assembly, stimulus-responsive release and synergistic integration with other therapeutic modalities such as photothermal therapy, photodynamic therapy, chemotherapy and immunotherapy via 2D/3D DNA architectures or organic–inorganic hybrid materials [50,51,52,53,54,55,56]. Meanwhile, new DNAzymes targeting complex transcriptomic species, including miRNAs, long non-coding RNAs (lncRNAs) and pseudogene transcripts, have continued to emerge, with increasingly prominent roles in early diagnosis, dynamic imaging and mechanistic studies [45,57,58].

**Figure 1:**
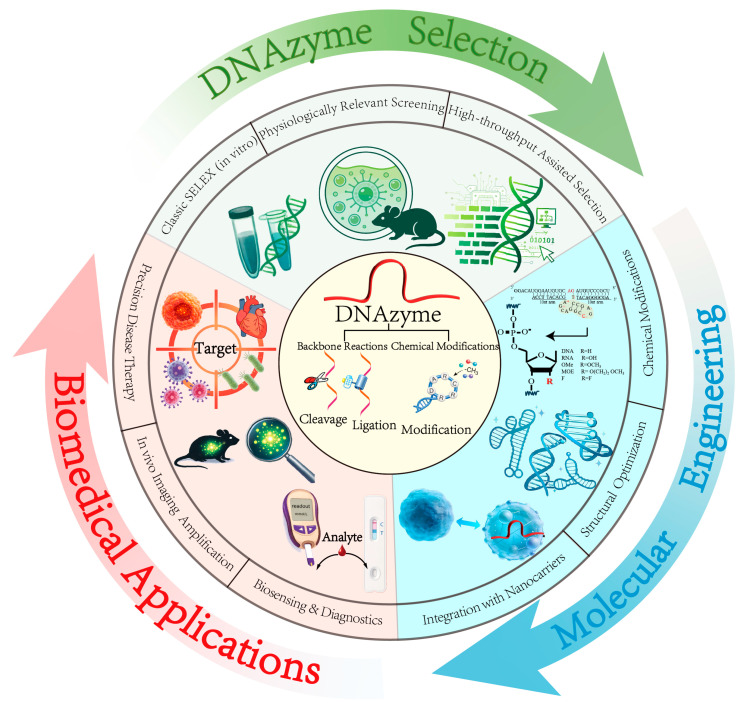
Schematic overview of DNAzyme research progress from selection strategy evolution to molecular engineering and biomedical applications. The center highlights core catalytic modes of DNAzymes, including cleavage, ligation and chemical modification. Surrounding modules summarize three major advances: (1) selection strategies from classical in vitro SELEX to physiologically relevant and high-throughput selection; (2) molecular engineering, including chemical modification, structural optimization and integration with functional nanocarriers; and (3) biomedical applications, spanning biosensing/diagnostics, in vivo imaging, signal amplification and precision therapeutic interventions.

Existing reviews have addressed therapeutic DNAzymes and DNAzyme-based biosensors, yet most are organized around specific applications or material platforms and do not provide an integrated synthesis spanning selection strategies, molecular optimization and biomedical implementation [59,60,61,62,63]. In particular, key questions remain insufficiently addressed from a mechanistic and design-oriented standpoint: how to pre-impose physiologically relevant constraints during selection, how to reconcile catalytic activity with in vivo stability through structural and chemical modifications and how to tailor application modalities across distinct disease models. Motivated by these gaps, this review establishes a unified design paradigm that treats the entire DNAzyme development process as a dynamic, feedback-driven cycle. As illustrated in the integrated workflow in Figure 1, We aim to construct an integrated landscape from molecular design to functional deployment, providing a clear roadmap for transforming empirical trial and error into rational, application-oriented molecular engineering. Specifically, we emphasize: (1) treating selection, enhancement, and application as a continuous design and iteration workflow; and (2) identifying practical design principles that balance catalytic performance with biocompatibility across various biological settings. To navigate this workflow, we systematically review selection strategies ranging from classical SELEX to cellular or in vivo selection and computation-assisted design, followed by a summary of enhancement approaches, including chemical modification and integration with nanocarriers. Finally, we survey representative advances in biosensing, in vivo imaging, and disease therapy, concluding with key challenges in mechanistic elucidation and preclinical evaluation.

## 2. Selection Strategies for DNAzymes

The discovery and optimization of DNAzymes rely heavily on in vitro evolution, with SELEX remaining the most central methodological route. With advances in high-throughput sequencing, microfluidics and computational modeling, DNAzyme selection has evolved from traditional multi-round enrichment into a more complex framework driven by multidimensional selection pressures, and is increasingly capable of coupling to physiological environments and collaborating with AI-assisted design [64,65]. This section systematically reviews the development of DNAzyme selection technologies from four perspectives: (i) classical SELEX, (ii) selection designs constrained by function and environment, (iii) high-throughput and emerging selection platforms, and (iv) structure- and computation-driven design.

### 2.1. Classical SELEX and Its Application in DNAzyme Discovery

In vitro evolution constitutes the core step for obtaining and optimizing DNAzymes, and its methodology can be traced back to the SELEX strategy first proposed in 1990 [66]. In 1994, Breaker and Joyce obtained the first catalytically active DNAzyme using SELEX, establishing the experimental framework for DNAzyme research [1]. While experiments typically begin with a standard randomized nucleic acid library, a fundamental distinction exists between aptamer and DNAzyme selection [64]. In contrast to aptamer SELEX, which enriches sequences primarily through affinity-mediated binding and retention, DNAzyme selection is fundamentally driven by a catalytic reaction, with active sequences recovered on the basis of product formation (e.g., substrate cleavage) rather than binding alone [67,68].

**Figure 2:**
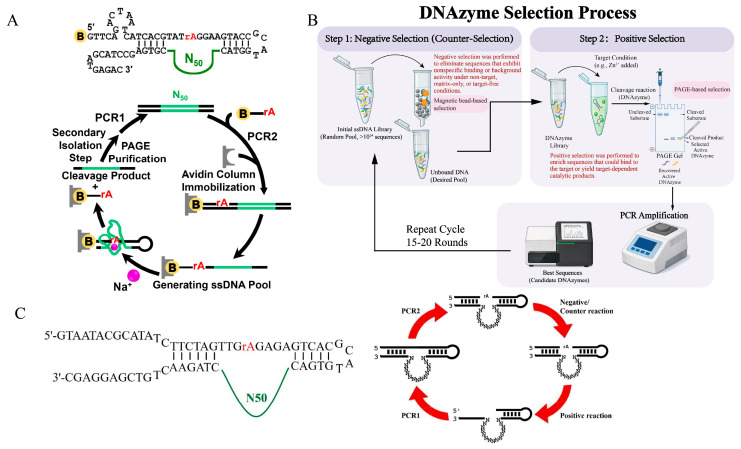
Schematic representation of in vitro selection strategies for RNA-cleaving DNAzymes. (**A**) In vitro selection of a Na^+^-specific DNAzyme. Reprinted with permission from Ref. [30]. Copyright 2015, National Academy of Sciences. (**B**) Negative-positive selection strategy. Certain visual elements in this diagram were generated using Google Gemini. (**C**) Schematic of the sequential negative-positive selection strategy for isolating high-specificity Mn^2+^-DNAzymes. Reprinted with permission from Ref. [9]. Copyright 2023, American Chemical Society.

To exploit this catalytic capability, the cleavage-induced elution strategy has been widely adopted. As illustrated by the selection of sodium-specific DNAzymes (Figure 2A), a biotinylated library is typically immobilized on a solid support. Upon addition of specific cofactors, functional sequences catalyze substrate cleavage and liberate themselves from the beads into the supernatant, thereby directly coupling physical separation with catalytic turnover [30]. Furthermore, to maximize specificity, contemporary protocols routinely integrate a counter-selection step preceding positive selection to deplete background or off-target sequences (Figure 2B). For instance, in the recent discovery of a highly selective Mn^2+^-DNAzyme (Figure 2C), the library was first challenged with high concentrations of interfering ions (e.g., Mg^2+^, Ca^2+^) to eliminate non-specific variants before triggering the specific reaction with Mn^2+^ [9]. This rigorous counter-selection prior to positive selection facilitates an enrichment trajectory driven predominantly by robust and target-specific catalytic activity.

**Figure 3:**
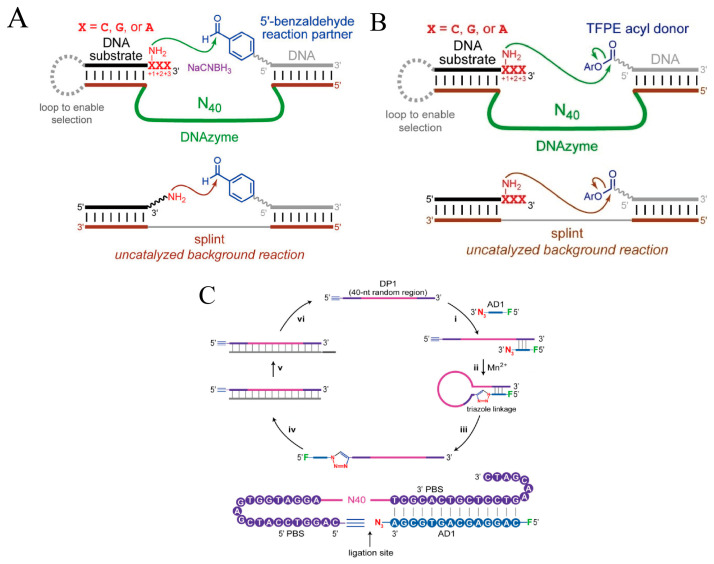
Selection strategies for DNAzymes catalyzing bond-forming reactions. (**A**) Selection design for DNAzymes catalyzing reductive amination. Reprinted with permission from Ref. [4]. Copyright 2024, Oxford University Press. (**B**) Selection strategy for amide bond-forming DNAzymes using an acyl donor. Reprinted with permission from Ref. [69]. Copyright 2024, Wiley-VCH. (**C**) Selection of DNA ligases forming triazole linkages via click chemistry, reprinted with permission from Ref. [3]. Copyright 2025, Oxford University Press.

Parallel to these methodological advancements, the catalytic repertoire of DNAzymes has expanded significantly beyond classical phosphodiester hydrolysis toward complex bond-forming and modification reactions (Table 2). For instance, Boyd et al. used PAGE-shift SELEX to isolate a DNAzyme that catalyzes site-specific N-alkylation on DNA nucleobases (Figure 3A), extending the reaction scope to post-synthetic nucleic acid modifications [4]. Similarly, by carefully controlling background reactions, Kennebeck et al. obtained DNAzymes that catalyze N-acylation of DNA or RNA bases (Figure 3B) with high positional specificity at selected C, G, and A sites [69]. For RNA methylation, Scheitl et al. evolved the methyltransferase ribozyme MTR1, enabling site-specific m^1^A installation [70]. In nucleic acid backbone ligation, Chang et al. reported the first DNAzyme capable of catalyzing a click reaction (CLDz2) (Figure 3C): uniquely using Mn^2+^ as a cofactor, it promotes inter-strand formation of a non-natural triazole linkage and enables highly selective synthesis of single-stranded circular DNA [3]. For signal readout, Volek et al. developed Apollon with a yellow colorimetric output and Supernova with a chemiluminescent output, further expanding the range of functions accessible by classical SELEX [71,72].

Despite the foundational success of classical SELEX, a critical bottleneck remains: the landscape of catalytic DNA is still disproportionately dominated by a few canonical scaffolds, most notably the 10-23 and 8-17 motifs. This scarcity of novel scaffolds can be attributed to several intrinsic and methodological factors. First, structurally, DNA lacks the 2′-hydroxyl group and the diverse functional side chains found in RNA and proteins, inherently limiting its capacity to form complex catalytic centers for difficult transformations [73]. Second, methodologically, classical SELEX introduces a survival of the simplest bias. During PCR amplification and enrichment, small, rapidly folding motifs (like the small bulge loops of 8-17) often outcompete larger, structurally complex, but potentially more versatile candidates [74,75,76]. Finally, insufficient sequence space coverage in traditional cloning-based characterization has likely left many rare but high-performance motifs undiscovered. Future opportunities, therefore, lie in leveraging deep-sequencing-assisted selection and machine learning to bypass these biases [48,77].

### 2.2. SELEX Designs Constrained by Function and Environment

Driven by the expanding utility of DNAzymes in biomedical sensing, disease diagnostics, and stimuli-responsive materials, selection strategies are transitioning from the optimization of isolated catalytic metrics toward application-driven directed evolution. Consequently, SELEX methodologies increasingly necessitate customized designs tailored to specific functional requirements, including cofactor selectivity and allosteric control, as well as diverse physiological or non-physiological environmental constraints.

**Figure 4:**
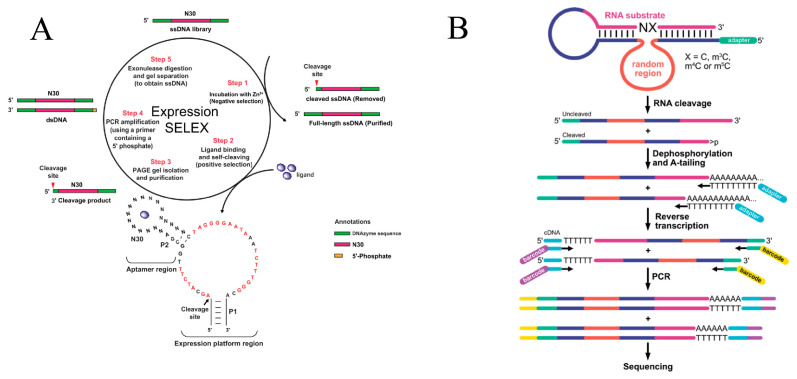
Advanced strategies for functional DNAzyme selection. (**A**) Expression SELEX strategy for developing ligand-dependent allosteric DNAzymes, reprinted with permission from Ref. [78]. Copyright 2023, Oxford University Press. (**B**) Workflow for high-throughput sequencing and activity analysis of DNAzyme pools. Reprinted with permission from Ref. [49]. Copyright 2022, American Chemical Society.

Consequently, the focus of library design and selection pressure has broadened from maximizing simple catalytic turnover to engineering precise allosteric regulation and cofactor selectivity. This shift allows for the isolation of DNAzymes that function as intelligent molecular switches or operate with non-canonical metal centers. To obtain high-performance allosteric DNAzymes, Chen et al. modified Expression-SELEX (Figure 4A) by introducing λ-exonuclease digestion and extending negative selection time, yielding a DNAzyme with high sensitivity to L-phenylalanine and extremely low background self-cleavage [78]. To mitigate reliance on a single metal-ion cofactor, Huang et al. incorporated chemical ligands such as imidazole into library design and isolated a DNAzyme exhibiting exponentially amplified selectivity for Zn^2+^, demonstrating that chemical modification can help overcome catalytic bottlenecks inherent to natural DNA [79].

Environment-constrained SELEX has also emerged as a major recent direction, aiming to conduct selections under target physiological conditions as well as under deliberately imposed non-physiological stresses. Conventional selections are often performed in idealized buffers, which can lead to substantial activity loss in real physiological settings. To further bridge the gap between in vitro activity and intracellular efficacy, a function-guided, cell-effective evolution/engineering paradigm has gained traction, in which DNAzyme chemotypes and architectures are optimized under cell-relevant constraints and validated by cellular functional readouts such as persistent mRNA knockdown in mammalian cells [43,51,80]. Furthermore, extending beyond physiological mimicry, Chang et al. developed DMSO-SELEX and isolated an RNA-cleaving DNAzyme that requires an organic cosolvent to function under conditions containing 35% DMSO, illustrating how specific environmental constraints can reshape catalytic properties [68]. In response to acidic microenvironments relevant to infection-associated settings, Zhou et al. obtained acidic RNA-cleaving DNAzymes (aRCDs) that operate efficiently at pH 5.3 and require only monovalent metal ions, partially overcoming the difficulty of maintaining DNAzyme activity under acidic conditions [81]. Furthermore, selection and engineering efforts toward biologically relevant cofactors have yielded DNAzymes responsive to ions such as Na^+^ for demonstrated intracellular sensing as well as redox-active Fe^2+^/Fe^3+^ to enable selective imaging in living systems [30,33]. Finally, pathogen-associated selection schemes that utilize pathogen-derived mixtures as activators have produced RNA-cleaving fluorogenic DNAzymes to recognize and report pathogenic bacteria, thereby enabling whole-cell or pathogen-associated detection frameworks [39,82].

### 2.3. High-Throughput and Emerging Selection Technologies

High-throughput sequencing and high-throughput sorting technologies have substantially transformed the efficiency and interpretability of DNAzyme SELEX [83]. HT-SELEX reduces dependence on validating only a few candidates and instead enables systematic analysis of enrichment landscapes and their dynamics across rounds. The DNAmoreDB database developed by Ponce-Salvatierra et al. integrates DNAzyme sequences and reaction conditions reported across hundreds of studies, providing a key resource for data-driven sequence mining and performance prediction [84].

Traditional SELEX often uses fold enrichment as a proxy for activity, which may miss low-abundance yet highly active sequences. To address this, Sednev et al. developed DZ-seq (Figure 4B), which directly reads the cleavage state of each sequence during sequencing, enabling parallel quantification of absolute catalytic activity for tens of thousands of sequences in a library [49]. Using a related concept, Streckerová et al. showed that for short-motif architectures, a single selection round combined with deep sequencing can rapidly identify high-performance RNA-cleaving DNAzymes, markedly shortening selection timelines [77]. Lozoya-Colinas et al. further extended this strategy to XNA aptamer discovery, achieving rapid development of functional nucleic acids via parallel selection across multiple chemotypes [85].

Microfluidic technologies provide compartmentalized, low-volume and high-throughput reaction platforms for DNAzyme selection [86]. Li et al. developed DNAzymes in droplets, encapsulating reactions in picoliter droplets and integrating microfluidic counting and sorting to screen DNAzymes responsive to pathogenic bacterial secreted proteins, achieving sensitivity down to the single-bacterium level [87]. The multiplexed fluorescence-activated droplet sorting platform established by Caen et al. offers a scalable tool for selecting multiple environment-responsive DNAzymes in parallel [88].

To introduce enhanced chemical functionality, Qin et al. developed SuFEx-SELEX, incorporating covalent warheads into libraries and successfully selecting aptamers capable of covalent crosslinking to target proteins. This framework also provides conceptual guidance for developing covalent catalytic DNAzymes [89]. Lee et al. employed AuNP color changes as a direct readout to establish visual AuNP-SELEX, enabling real-time monitoring of selection progress and optimization of conditions [90].

### 2.4. Structure-Guided and Computation-Assisted DNAzyme Design

With advances in high-resolution structural determination and molecular simulation, DNAzyme discovery is transitioning from empirical blind selection enrichment toward a more rational paradigm of structure-driven design, prediction-assisted selection and experimental validation [91,92].

Early understanding of the 10-23 DNAzyme relied largely on biochemical assays; more recently, Cramer et al. resolved its crystal structure [41], and Borggräfe et al. used time-resolved NMR to reveal dynamic conformational changes during its catalytic cycle, providing direct structural bases for site-specific modification and optimization [40]. Guided by structural principles, Shi et al. optimized a locking-arm-recognition-loop module to design a programmable single-stranded allosteric DNAzyme, enabling highly sensitive nucleic acid detection without amplification [93]; Wu et al. further adopted a modular strategy to construct a membrane-anchored DNAzyme molecular machine for cascading regulation of intercellular signaling [94]. In materials integration, both the self-contained covalent G4-hemin DNAzyme Co-G4N/H designed by Li et al. and Coronazyme developed by Zuo et al. substantially enhanced the peroxidase-like activity of G4-hemin DNAzymes through spatial structural engineering [95,96].

Conventional computation-assisted design has primarily relied on secondary-structure energy models and simple statistical learning. For example, Pine et al. used logistic regression to predict 10-23 cleavage efficiency based on sequence and structural features [97]. In recent years, deep learning has begun to gain traction in the DNAzyme field. The SequenceCraft platform developed by Eremeyeva et al. applies machine-learning models to predict rate constants of RNA-cleaving DNAzymes given sequence features and experimental conditions, demonstrating the feasibility of data-driven selection and optimization [98]. Despite the relative paucity of high-quality DNAzyme data compared to protein enzymes, architectures like DeepEnzyme herald a future of precise engineering. An iterative cycle of computational generation, experimental selection and data feedback facilitates the creation of DNAzymes tailored to specific substrates and environments [99].

## 3. Molecular Engineering of DNAzymes

Although multidimensional selection strategies can endow DNAzymes with defined catalytic activities and a degree of environmental adaptability, naked nucleic acid sequences still face substantial physiological barriers when translated from in vitro systems to in vivo settings, including rapid nuclease-mediated degradation, inefficient cellular uptake and potential off-target effects [100,101]. Therefore, converting the catalytic potential obtained by selection into practical therapeutic efficacy typically requires post-selection engineering [102,103]. This section summarizes strategies to enhance DNAzyme performance from three perspectives: chemical modifications to improve stability, structural engineering to modulate catalytic behavior and nanocarrier-enabled targeted delivery.

### 3.1. Chemical Modifications to Improve Stability and Affinity

Chemical modification has increasingly become a technical route that simultaneously supports kinetic optimization and adaptation to physiological environments. In general, commonly used modifications can be categorized into five classes, with an additional emerging direction: (i) sugar/2′-position (conformational) modifications, (ii) phosphate backbone modifications, (iii) nucleobase and functional side-chain modifications, (iv) terminal protection and conjugation, and (v) mixed XNA chemical evolution, together with post-synthetic site-specific functional installation.

**Figure 5:**
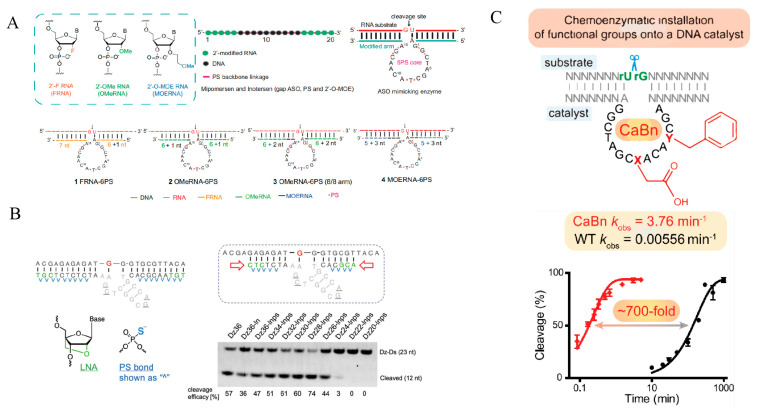
Strategies for the chemical optimization of DNAzyme. (**A**) Optimization of modification patterns in the substrate-binding arms of 10-23, reprinted with permission from Ref. [104]. Copyright 2025, Oxford University Press. (**B**) Arm optimization of an 8-17-derived DNAzyme using ASO-inspired chemistries, reprinted with permission from Ref. [42]. Copyright 2023, The Royal Society of Chemistry. (**C**) Post-synthetic side-chain installation to enhance DNA catalytic activity, reprinted with permission from Ref. [110]. Copyright 2024, American Chemical Society.

Among these, sugar (2′-position/conformational) modifications have become one of the key approaches for improving DNAzyme turnover efficiency. Using 10-23 as a parental scaffold, the Chaput group performed systematic scanning by introducing a high fraction of XNA sugars (e.g., FANA/TNA) into the binding arms and limited point substitutions within the catalytic core; the resulting X10-23 substantially improved the balance between substrate binding and product release, increased the population of active conformations and enabled high-turnover cleavage under physiological Mg^2+^ conditions [80]. Building on this, Dz46 further refined core determinants (e.g., G14) that prefer 2′-MOE, 2′-OMe, or 2′-F substitutions and combined ASO-like modifications in the recognition arms, yielding one of the most active 10-23 variants reported under physiological conditions [51]. More recently, the Chaput laboratory integrated 2′-F, 2′-OMe, 2′-MOE with PS modules (Figure 5A) to map relationships among modification type-site distribution-kinetic parameters, highlighting that moderately increasing affinity while avoiding product inhibition is critical for improving catalytic efficiency [104]. In the 8-17 system, X8-17 incorporated 2′-modifications/LNA/PS in the arms together with sparse XNA substitutions in the core (Figure 5B), maintaining effective intracellular cleavage activity and suggesting that this strategy can be generalized beyond the 10-23 scaffold [42]. Consistently, methylation within the catalytic core (5 mC/m^6^A) markedly suppresses 8-17 activity, whereas arm modifications exert smaller effects, underscoring the high sensitivity of the catalytic core to its local chemical microenvironment [105]. A 2′-C-methyl nucleoside scan further indicated a narrow tolerance window for sugar modifications in the 8-17 core, necessitating coordinated optimization with arm modifications [106].

Phosphate backbone modifications typically enhance performance indirectly by improving biostability and tuning metal-ion compatibility. Classical phosphorothioate linkages have been widely adopted as stabilization modules in X10-23, Dz46, and ASO-like DNAzymes [51,80,104]. More advanced backbone chemistries, such as mesyl phosphoramidate (MsPA), phosphoramidate/phosphoramidate ester (PN/PG) and LNA-sulfamate/sulfamide, have been shown in the ASO field to markedly increase nuclease resistance and modulate protein binding and immune profiles; however, systematic performance evaluation in DNAzyme contexts remains limited, leaving a clear gap for establishing connections between backbone engineering and catalytic output [107,108]. Notably, synthetic methodologies now enable the introduction of phosphoramidate-type linkages and the installation of further functionalizable chemical handles during solid-phase synthesis, providing a technical foundation for DNAzyme backbone functionalization [109].

Nucleobase-level and functional side-chain modifications can directly modulate metal selectivity and the catalytic microenvironment. Yu Hanyang’s group developed a post-synthetic AP-site-oxyamine coupling strategy in which an abasic site is pre-positioned within the catalytic core and small-molecule side chains are installed via oxime formation; dual-site cooperative modification at T8 and A12 in the 10-23 core (Figure 5C) produced nearly an order-of-magnitude activity enhancement. This approach is also applicable to other scaffolds such as 8-17 and 9DB1, indicating broad potential for cross-scaffold side-chain engineering [110]. In addition, SELEX using random libraries doped with modified nucleosides has yielded DNAzymes capable of catalyzing new reaction types such as nucleobase reductive amination, illustrating that chemical expansion can not only optimize performance but also facilitate the discovery of new scaffolds and catalytic repertoires [4].

Terminal protection and PEG/lipid conjugation are primarily deployed for in vivo applications, where they suppress exonuclease degradation, prolong circulation time and enhance cellular uptake to increase the effective catalytic dose of DNAzymes; these measures are often combined with sugar and backbone optimization strategies described above [16].

Taken together, current DNAzyme enhancement increasingly follows a combinatorial modification paradigm: 2′-sugar and XNA elements primarily tune the kinetics of substrate binding/product release, PS and terminal/conjugation modifications improve stability and exposure time in physiological environments, whereas catalytic core functionalization and metal microenvironment engineering directly influence k_cat_ and catalytic selectivity. Coordinated division of labor and synergy across these strategies should progressively transform in vitro-evolved DNAzymes into efficient, stable and translatable catalysts under physiological conditions.

### 3.2. Structural Engineering and Sequence Optimization

After establishing baseline chemical stabilization, further improvements in DNAzyme specificity and controllability largely rely on engineering of secondary structures and modular architectures. Unlike bulky protein enzymes, DNAzyme catalytic cores are typically composed of only a few dozen nucleotides and are highly sensitive to surrounding sequences and spatial context. Consequently, recognition arm length and composition, reconfigurable secondary-structure frameworks and multivalent organization provide practical handles for fine control of catalytic behavior.

The most direct structural engineering approach is to tune the length and base composition of the recognition arms to optimize affinity for the target sequence and mismatch tolerance. Arms that are too short destabilize the complex and reduce effective catalytic frequency, whereas overly long arms can over-stabilize the DNAzyme–substrate complex, hinder product release and manifest as reduced apparent turnover. Systematic variation in GC content, mismatch positions and mismatch numbers allows precise modulation of melting temperature and binding free energy across different target contexts, improving discrimination of single-nucleotide differences and enabling high-precision identification of SNPs or splice variants. Such arm engineering is commonly implemented together with 2′-modifications, LNA, or PS to achieve a better overall balance among stability, affinity and specificity [111,112,113].

**Figure 6:**
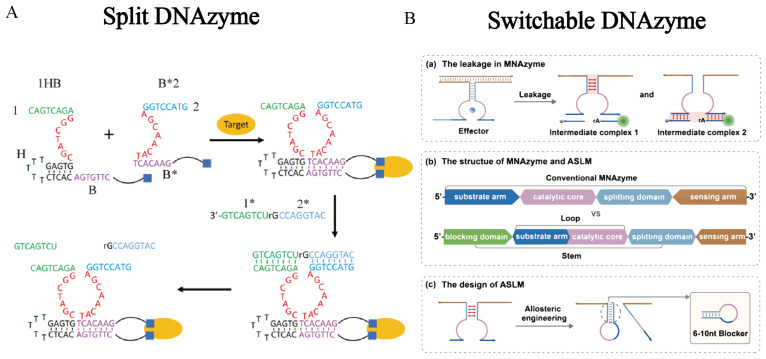
Representative activation mechanisms of DNAzymes. (**A**) Protein-induced proximity assembly of a split DNAzyme: target binding colocalizes two inactive subunits, promotes assembly and restores catalytic activity. Reprinted with permission from Ref. [114]. Copyright 2021, American Chemical Society. (**B**) Allosteric hairpin lock-triggered unlock switchable DNAzyme: sequestration of functional domains suppresses background leakage and enables input-dependent activation, reprinted with permission from Ref. [115]. Copyright 2024, Elsevier B.V.

Building on arm tuning, reconfigurable secondary structures have been widely used to construct switchable and split DNAzymes (Figure 6). In switchable designs, the catalytic core is partially sequestered within a hairpin, G-quadruplex or other folded structure, and is exposed only upon a defined input via structural rearrangement, enabling digital-like responses to environmental cues. Split DNAzymes divide the catalytic core into two or more oligonucleotide fragments that are catalytically inactive in isolation, but reassemble into an active structure only when bridged by a target molecule or brought into proximity by spatial clustering. These designs convert DNAzymes from constitutively active scissors into environmentally responsive molecular switches, well-suited for constructing responsive biosensors and programmable molecular logic gates [114,115].

Beyond switchable and split architectures, the construction of multivalent and multisite DNAzymes further expands functional boundaries. By rationally concatenating or logically connecting multiple DNAzyme units, researchers can enable cascade amplification, multi-target coupling and logical computation. For example, linearly or circularly linking multiple RNA-cleaving DNAzymes can allow sequential cleavage of multiple substrates after a single trigger, amplifying signal output; arranging DNAzymes with different specificities in AND or OR configurations enables combinatorial recognition of complex molecular patterns. Consequently, DNAzymes are no longer isolated catalytic components but can be assembled into molecular circuits, providing a highly programmable platform for multiplexed diagnostics and precision interventions [116,117,118].

### 3.3. Nanocarriers and Scaffolds for Amplifying DNAzyme Performance

Despite chemical and structural optimization, free DNAzymes in complex biological environments often still suffer from low effective local concentration, susceptibility to nuclease degradation, and limited ability to localize precisely to target sites. Immobilizing or assembling DNAzymes on nanocarriers, ranging from inorganic nanoparticles to self-assembled DNA nanostructures, can markedly increase their stability and local concentration both in vitro and in vivo. Moreover, multivalency and spatial organization provided by these scaffolds can amplify apparent catalytic performance, while offering additional design freedom for signal transduction and functional integration [119,120].

Inorganic carriers, including gold nanoparticles, quantum dots, metal–organic frameworks and magnetic nanoparticles, provide size-tunable, readily functionalized and high-density loading platforms for DNAzymes [121,122,123]. Gold nanoparticles, for example, enable multivalent covalent grafting of DNAzymes through Au-S linkages, improving resistance to degradation and enhancing apparent catalytic efficiency via multivalent binding and local enrichment; their plasmonic properties further support the construction of colorimetric or fluorescence/quenching readout schemes [124,125]. Similarly, MOFs and covalent organic frameworks, owing to their high surface areas and tunable pore environments, can efficiently load DNAzymes and, through metal-ion enrichment or substrate preconcentration, enable highly sensitive in vitro detection of analytes such as Pb^2+^ and miRNAs, highlighting their potential in catalytic sensing and diagnostics [126,127].

Compared with inorganic carriers, self-assembled DNA nanostructures (e.g., DNA origami and DNA tiles) offer more precise spatial programmability. By anchoring multiple DNAzyme units at predefined positions, rigidly constrained multi-enzyme arrays can be constructed to process substrates sequentially or in parallel at the nanoscale. For example, hexagonal DNA nanocages or multibranched DNA architectures can display multiple copies of RNA-cleaving DNAzymes on internal or external surfaces to improve intracellular cleavage efficiency. DNA walker systems can also use target miRNAs as initiation signals to drive DNAzyme-carrying walkers along tracks, achieving programmable molecular amplification through iterative cycles of walking–cleavage–signal gain. These designs tightly couple single-molecule catalytic function with nanoscale spatial organization, laying the groundwork for smart nucleic acid nanomachines with spatiotemporally controllable behaviors [128,129].

In addition, embedding DNAzymes into soft materials such as hydrogels, liposomes and biomimetic membranes provides a route for translating nanoprobes toward macroscopic devices or implantable/wearable systems. DNAzyme-crosslinked hydrogels can undergo degradation or swelling in response to physiological stimuli, enabling on-demand release of drugs or signal probes. Injectable DNAzyme-based hydrogels can form localized, high-concentration reaction compartments in the tumor microenvironment for sustained signal amplification or therapeutic cleavage. Further, integrating DNAzymes with biomimetic membranes or microneedle patches can enable localized transdermal or transmucosal delivery, maintaining high local efficacy while reducing systemic exposure and associated adverse effects. Overall, nanocarriers and spatial scaffolds not only enhance the stability and apparent catalytic performance of individual DNAzyme molecules but also provide a multi-level design space, from molecular constructs to functional devices for integrated applications in complex biological settings [53,130,131,132,133,134].

Overall, chemical modification, structural engineering, nanoscaffolds and delivery systems together constitute a bottom-up, multi-level design framework from fine structural control at the nucleotide level to molecular-scale optimization of DNAzyme catalysis and further to nanoscale spatial organization as well as in vivo targeted delivery and subcellular localization. An increasing body of evidence suggests that only through coordinated design across these layers and balancing catalytic activity, biostability, safety, and practicality of administration can DNAzymes progress from highly efficient molecular scissors in vitro to nucleic acid therapeutic platforms with genuine translational and clinical potential.

### 3.4. Challenges and Failure Modes

While the engineering strategies discussed above have yielded significant successes, reporting negative results and failure modes is equally critical for the field’s maturity [80,101]. First, chemical modification faces steep activity cliffs: unlike the tolerant recognition arms, the catalytic core is highly sensitive, where even subtle nucleobase/backbone modifications at conserved core positions can severely compromise activity (e.g., 2′-C-methyl, LNA, or site-specific backbone caging) [106,135,136]. Second, environmental incompatibility remains a major hurdle; DNAzymes selected in simplified buffers frequently fail in vivo due to low free Mg^2+^ availability or competitive inhibition by Ca^2+^ and cellular proteins, emphasizing the need for the rigorous counter-selection strategies mentioned in Section 2.2 [29,80]. Finally, nanocarrier integration can be counterproductive if linker designs are too short or loading densities are too high, which may induce steric hindrance or prevent the conformational switching required for catalysis [40,119]. Acknowledging these pitfalls helps avoid redundant trial-and-error and guides a more rational design.

## 4. Advances in the Biomedical Applications of DNAzymes

Benefiting from the establishment of high-throughput, efficient selection platforms described in the preceding sections, together with advances in performance enhancement strategies such as chemical modification and nanoscale assembly, DNAzymes have achieved markedly improved catalytic efficiency and biostability. These gains enable DNAzymes to retain functionality in more complex physiological milieus, thereby laying a solid foundation for their transition from laboratory-based fundamental research to clinically relevant applications. Against this backdrop, this section concentrates on the practical performance of high-performance DNAzyme systems in translational medicine. Along a progressive trajectory from high-sensitivity molecular diagnostics to spatiotemporally controlled in vivo imaging and precision disease intervention, we systematically summarize representative applications and emerging advances of DNAzymes across the biomedical pipeline.

### 4.1. Applications of DNAzymes in Biosensing and Molecular Diagnostics

As a class of programmable functional nucleic acids, DNAzymes combine sequence-level designability with enzyme-like catalytic activity and have consequently enabled a multilayered landscape of applications in biosensing and molecular diagnostics. Broadly, their use in detecting metal ions and small molecules, profiling nucleic acid and protein biomarkers and powering portable point-of-care testing platforms together constitutes a continuous translational pipeline from laboratory assays to bedside implementation.

In the domain of sensing metal ions and small molecules, DNAzymes responsive to metals such as those dependent on Pb^2+^, Mg^2+^, and Zn^2+^ function as intrinsically selective recognition elements for constructing highly specific sensors [137,138,139,140]. These DNAzymes typically rely on metal-ion-triggered self-cleavage or substrate-strand scission, thereby transducing otherwise difficult-to-monitor ionic concentrations into measurable fluorescence, colorimetric or electrochemical outputs [130,141,142,143]. Such sensor designs have been applied to the detection and semi-quantitative assessment of metal-ion levels in complex biological matrices, including serum and urine, providing practical tools for evaluating disruptions in metal-ion homeostasis and their potential biological consequences [144,145,146,147].

Nevertheless, accurate metal analysis in complex biofluids is often more challenging than protein or nucleic acid detection due to strong buffering, protein binding and matrix interference. Consequently, many clinically deployed biomarker panels emphasize nucleic acid fragments and proteins [148,149,150,151,152]. To broaden their applicability, DNAzymes are increasingly incorporated into diverse signal amplification architectures as catalytic amplification modules rather than direct recognition elements [151,152]. In nucleic acid assays, target sequences are typically recognized with high specificity via base pairing or an aptamer, which subsequently initiates amplification networks that integrate DNAzyme modules [148,153]. For instance, isothermal strategies independent of protein enzymes, such as hybridization chain reaction (HCR) and catalytic hairpin assembly (CHA), have evolved from simple assembly mechanisms to sophisticated catalytic cascades. In these systems, a single target binding event triggers the self-assembly of metastable hairpins into long, nicked polymers that display numerous active DNAzyme units, thereby achieving exponential signal gain through the coupling of assembly kinetics and enzymatic turnover [154,155,156,157,158]. By contrast, enzymatic amplification approaches such as rolling circle amplification rely on polymerases to generate long repetitive strands, onto which multiple DNAzyme motifs can be loaded, achieving dual amplification through sequence replication coupled with multivalent site loading [58,159,160,161]. Furthermore, the field has witnessed the rise in dynamic DNA nanomachines, specifically DNAzyme walkers [162,163]. Unlike static probes, these walkers autonomously traverse substrate-modified tracks (e.g., on nanoparticles or cell surfaces), cleaving multiple substrate strands sequentially to generate continuous signal outputs [164]. This walking mechanism effectively transforms the detection of low-abundance targets, such as miRNAs, into easily measurable signals through prolonged, autonomous catalytic cycles [165]. With appropriate design, these amplification modules are not only suitable for quantitative analysis of low-abundance nucleic acids but can also be extended to protein targets such as tumor biomarkers and inflammatory factors via aptamer- and protein-binding interfaces [148,149,150,166,167].

Beyond signal amplification, the integration of DNAzymes with molecular logic gates represents a significant leap toward intelligent diagnostics [168]. By coupling catalytic activity with Boolean logic operations (such as AND, OR, and NAND), DNAzyme sensors can process multiple molecular inputs simultaneously to generate a consolidated output [116,169]. This capability is particularly vital for precision medicine, where a single biomarker often lacks sufficient specificity. For example, AND gate sensors are designed to trigger signal release only when two distinct biomarkers are present, effectively acting as a coincidence filter to minimize false positive rates in complex biological matrices [170]. Recent advances have further expanded this concept to constructing autonomous molecular circuits that can perform sequential logic operations, enabling the precise discrimination of cell subtypes or viral variants based on multi-input signatures [171,172].

**Figure 7:**
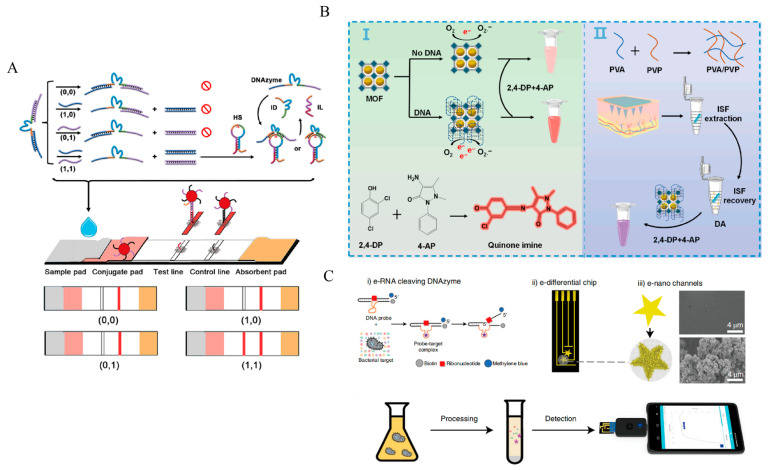
Integration of DNAzymes into various portable and visual diagnostic platforms. (**A**) DNAzyme-integrated lateral flow assay (LFA) for visual disease diagnosis. Reprinted with permission from Ref. [175]. Copyright 2024, Elsevier B.V. (**B**) Laccase-like DNAzyme@MOF platform for biomarker detection in interstitial fluid. Reprinted with permission from Ref. [125]. Copyright 2025, Elsevier B.V. (**C**) DNAzyme-integrated handheld electrochemical chip for point-of-care testing. Reprinted with permission from Ref. [177]. Copyright 2021, Springer Nature.

As molecular diagnostics continues to expand toward point-of-care and at-home testing, DNAzymes are increasingly being embedded into a range of portable and visual readout platforms. Paper-based sensors exploit capillary wicking within cellulose fibers to enable passive fluid transport, immobilize DNAzyme-functionalized probes in the test zone, and translate analyte-triggered reactions into colorimetric or fluorescent outputs for naked-eye inspection or quantitative readout using handheld devices (Figure 7A). Relative to conventional lateral flow strips using protein antibodies, DNAzyme-enabled paper platforms offer superior environmental robustness without requiring cold-chain storage. Furthermore, compared to enzyme-free amplification circuits (e.g., HCR), which rely on slow thermodynamic equilibrium, the catalytic turnover of DNAzymes provides rapid signal kinetics essential for time-critical POC scenarios [173,174,175]. Beyond paper substrates, DNAzymes have been integrated into microneedle patches and wearable devices to enable minimally invasive sampling of interstitial fluid or sweat (Figure 7B), supporting continuous monitoring of analytes such as electrolytes and toxic metal ions; importantly, their nucleic acid nature also facilitates monolithic fabrication with flexible substrates and microfluidic architectures [125,176]. Moreover, coupling DNAzyme readouts with smartphones or portable readers (Figure 7C), leveraging phone cameras and dedicated applications for signal acquisition and data processing, may further propel point-of-care testing toward digitalized, network-enabled care paradigms [177,178,179,180,181].

Overall, the role of DNAzymes in biosensing and molecular diagnostics has evolved from serving as single-purpose metal-ion probes to acting as broadly applicable modules for signal amplification and transduction across diverse reaction modalities and assay platforms. Although challenges remain, particularly with respect to stability in complex matrices and safety considerations for in vivo applications, the maturation of these diagnostic strategies has established a critical foundation for leveraging DNAzymes in broader biomedical contexts, including subsequent in vivo imaging and therapeutic intervention.

### 4.2. DNAzymes in In Vivo Imaging and Smart Probes

In the arenas of in vivo imaging and smart probe engineering, DNAzymes offer a distinctive advantage through activation-type signal generation enabled by the coupling of programmable recognition with catalytic reactivity. Because catalytic activity is unleashed only after a specific molecular event, background fluorescence can be markedly suppressed, thereby improving the signal-to-noise ratio for imaging at the cellular and even whole-animal levels. This switchable behavior is particularly well-suited for detecting trace targets in complex physiological environments, and it also provides a conceptual framework for constructing multimodal imaging platforms and integrated theranostic systems.

For activatable fluorescent probes, a common design principle is not simply to maximize fluorescence intensity, but rather to translate DNAzyme catalysis into an observable optical change via a catalytic beacon. Upon substrate cleavage, the spatial relationship between the quencher and the fluorophore is reconfigured, switching the signal from an off to an on state [166,182,183]. Although specific architectures vary in structural details, they share a unifying logic: integrating recognition (e.g., metal ions, miRNA, or mRNA), catalysis and signal amplification into a continuous reaction pathway. Consequently, these probes often achieve a favorable balance of specificity and sensitivity in applications such as monitoring perturbations in ion homeostasis and detecting aberrant tumor-associated transcripts [184,185,186]. Notably, in vivo performance remains constrained by probe stability and delivery efficiency. Nucleic acid degradation, endosomal sequestration and interference from local ionic microenvironments can all give rise to false negative or false positive readouts. Specifically, the intrinsic dependence on metal cofactors, which fluctuate in pathological states, renders DNAzymes less robust than small-molecule probes for absolute quantification, necessitating ratiometric designs that self-calibrate against environmental variability [187,188,189].

To address the limited tissue penetration and quantitative capability of standalone fluorescence imaging, multimodal imaging platforms have attracted increasing attention in recent years [190]. By hybrid assembly with MRI, CT or PET probes, DNAzymes can function as conditional trigger modules, whereas metal chelates, radionuclides or high-atomic-number materials provide advantages in deep-tissue visualization and quantitative analysis [191,192,193]. In parallel, carriers such as gold nanoparticles, iron oxide nanoparticles and upconversion nanoparticles have been employed to co-integrate fluorescence, photoacoustic and magnetic resonance signals, while programmable responsiveness is imparted through surface DNA assembly [194]. Within this framework, optical imaging offers rapid indication and high-resolution localization, whereas modalities such as MRI/PET enable whole-body biodistribution assessment and treatment-response monitoring; consequently, structured assembly coupled with complementary signal readouts is emerging as a key route to improving the translational potential of imaging systems.

In theranostic systems, the DNAzyme-enabled capacity for sensing followed by feedback-controlled drug release is further amplified. When lesion-associated molecular events such as ionic imbalance, aberrant expression of specific RNAs or characteristic features of the tumor microenvironment are detected, gated drug carriers are triggered to unlock, enabling on-demand release [195,196,197,198,199]. In parallel, imaging signals can report the extent of release in real time, thereby establishing an adaptive closed-loop regulation scheme [200]. In principle, this strategy may support individualized interventions that automatically tune treatment intensity according to lesion activity, but its reliability depends on biologically rational trigger thresholds, matching in vivo kinetics, and long-term safety evaluation. The evolution of DNAzyme imaging and smart probes is shifting from singular detection paradigms toward multiplexed validation integrated with feedback intervention, emphasizing the need for advanced delivery strategies, in vivo calibration, and clinical translation standards [201,202,203,204].

### 4.3. DNAzymes in Disease Therapy

**Figure 8:**
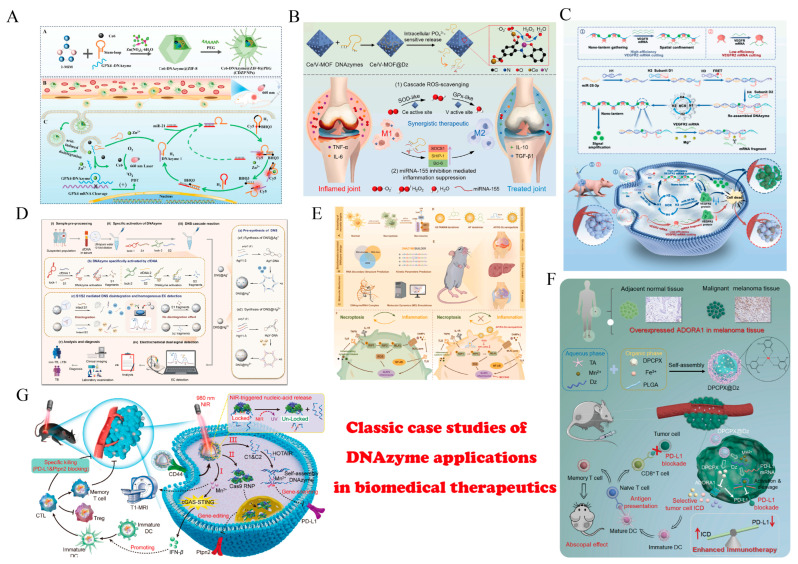
Representative therapeutic applications of DNAzymes in biomedicine. (**A**) GPX4-targeting CDZP NPs for potentiating PDT in breast cancer. Reprinted with permission from Ref. [195]. Copyright 2025, American Chemical Society. (**B**) Ce/V-MOF@Dz integrating antioxidant nanozymes and anti-miRNA-155 DNAzymes for rheumatoid arthritis. Reprinted with permission from Ref. [222]. Copyright 2025, Elsevier Inc. (**C**) miRNA-triggered self-assembly activating DNAzymes to cleave VEGFR2 mRNA for cancer therapy. Reprinted with permission from Ref. [209]. Copyright 2025, Elsevier B.V. (**D**) Electrochemical TB diagnosis via cfDNA-activated DNAzymes and ion-functionalized DNA nanosphere cascades. Reprinted with permission from Ref. [147]. Copyright 2025, Elsevier B.V. (**E**) RIP3 mRNA-targeting DNAzyme suppressing NLRP3 inflammasome-driven necroinflammation. Reprinted with permission from Ref. [221]. Copyright 2025, Elsevier B.V. on behalf of the Chinese Pharmaceutical Association. (**F**) Co-delivery of anti-PD-L1 DNAzyme and 1-MT to remodel the immunosuppressive microenvironment and enhance antitumor immunity. Reprinted with permission from Ref. [214]. Copyright 2023, Elsevier Ltd. (**G**) NIR-triggered logic nanomachines targeting PD-L1 mRNA for cancer immunotherapy. Reprinted with permission from Ref. [202]. Copyright 2025, Chinese Pharmaceutical Association and Institute of Materia Medica, Chinese Academy of Medical Sciences.

For therapeutic applications, RNA-cleaving DNAzymes achieve post-transcriptional regulation by specifically recognizing and cleaving target mRNAs. Owing to their high programmability, relatively efficient catalysis and engineerable chemical stability, DNAzymes have become important molecular tools in gene therapy. Across diverse disease models, their development has advanced from early proof-of-concept studies toward more systematic in vivo evaluation and exploration of combination therapies (Figure 8).

In oncology, DNAzymes primarily target oncogenic transcripts that drive tumor initiation and progression, such as c-myc, Bcl-2 and VEGF. By downregulating signaling pathways associated with proliferation, apoptosis resistance and angiogenesis, DNAzymes have shown antitumor effects in multiple solid and hematologic tumor models [104,183,205,206,207,208,209]. However, silencing a single gene is often insufficient to address the redundancy and plasticity of tumor signaling networks [210,211]; accordingly, recent studies emphasize combining DNAzymes with chemotherapy, radiotherapy or immunotherapy. Available evidence indicates that DNAzymes can increase tumor sensitivity to cytotoxic drugs or irradiation by reducing expression of resistance-associated genes [212,213]; DNAzymes targeting VEGF or immunosuppressive factors may also improve the tumor vascular and immune microenvironments, indirectly enhancing responses to immunotherapy [214]. These findings suggest that, in cancer settings, DNAzymes may be better positioned as sensitizers or regulatory adjuncts rather than as standalone therapeutics.

A rapidly emerging frontier in cancer therapy is the use of DNAzymes to modulate cell–cell interactions and the tumor microenvironment, extending their function beyond intracellular gene silencing [215,216]. Unlike traditional approaches that solely target internal oncogenes, recent strategies deploy membrane-anchored or secreted DNAzymes to intervene in intercellular signaling cascades [217]. For instance, Wu et al. constructed a membrane-anchored DNAzyme molecular machine that can be mechanically triggered to regulate signaling between adjacent cells, effectively programming intercellular communication [94]. In the context of immunotherapy, DNAzymes targeting immune checkpoints such as PD-L1 have been shown to remodel the immune synapse [197]. By silencing PD-L1 expression, these DNAzymes alleviate the brakes on T cells, thereby restoring potent anti-tumor immunity through enhanced T cell-tumor cell engagement [94,214]. This shift towards regulating the sociology of cell communities represents a sophisticated evolution in DNAzyme therapeutics.

In cardiovascular and metabolic diseases, DNAzyme research has focused more on abnormal vascular smooth muscle cell proliferation and inflammatory regulation. DNAzymes targeting cell-cycle regulators or pro-inflammatory cytokines can suppress vascular remodeling and neointimal hyperplasia in atherosclerosis and vascular injury models, indicating potential value for intervening in vascular lesions [218,219]. In infectious and inflammatory diseases, DNAzymes offer the advantage of directly targeting conserved viral genomic regions or key transcripts, thereby reducing viral replication burden; DNAzymes targeting central inflammatory mediators such as NF-κB and TNF-α have also been shown to significantly alleviate inflammation in multiple animal models [220,221,222].

In neurological and other disease areas, therapeutic exploration of DNAzymes remains comparatively early-stage. Based on the mechanism of sequence-specific mRNA cleavage to modulate protein expression, DNAzymes have been proposed as potential tools to intervene in the production of pathological proteins implicated in neurodegeneration, such as APP and α-synuclein, although systematic and mature experimental validation is still limited [223,224,225,226,227,228]. In addition, for ophthalmic and respiratory diseases, local administration represents a disease context best suited to DNAzyme kinetics. By delivering high concentrations directly to the target surface, this strategy effectively bypasses the systemic clearance and endosomal escape bottlenecks that severely handicap DNAzymes compared to lipid-conjugated siRNAs or ASOs, enhancing accumulation in target tissues while reducing systemic toxicity [229,230,231,232,233]. For example, Norbert Krug’s team developed a GATA-3-specific DNAzyme (SB010) targeting Th2-type inflammatory responses and delivered it locally by inhalation in asthma. Clinical and preclinical studies suggest that this strategy can significantly attenuate both early and late phase asthmatic responses and reduce associated inflammatory markers, highlighting the advantages of local DNAzyme delivery for improving targeting and safety [234,235,236]. Meanwhile, preclinical long-term inhalation studies and Phase I dose escalation trials of hgd40/SB010 have demonstrated good overall tolerability. Mechanistic evaluations further indicate no apparent non-specific innate immune activation, providing direct evidence for the low immunogenicity/immune stimulatory potential of DNAzymes [28,236,237]. Beyond allergic asthma, inhaled SB010 has been extended to COPD populations with elevated sputum eosinophils in randomized, double-blind and placebo-controlled feasibility studies [238]. Another line of work, motivated by the role of human rhinovirus as a key trigger of asthma exacerbations, designed and screened large panels of anti-HRV DNAzyme candidates targeting conserved viral RNA regions [239]. Overall, therapeutic applications of DNAzymes across diseases are increasingly shifting from single-target inhibition toward integrated strategies that combine combination therapy with microenvironment modulation.

## 5. Outlook

**Figure 9:**
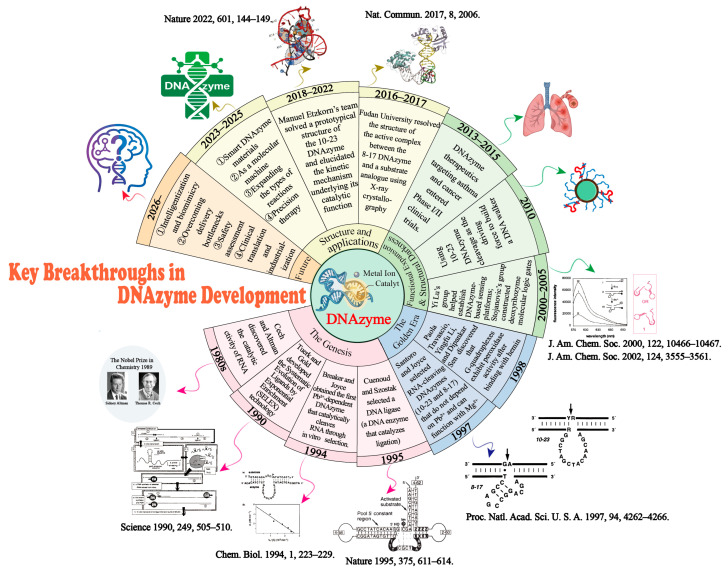
Timeline of key breakthroughs and future trajectories in DNAzyme development. The spiral timeline illustrates the five evolutionary stages of the field, reflecting the transition from fundamental discovery to clinical translation. (1) Retrospective (1980s–2025): Guided by a strategic progression from efficient selection to molecular engineering and subsequent biomedical applications, the field has witnessed the genesis of the first DNAzymes (1994) and the iconic 10-23/8-17 variants (1997), followed by functional expansion into biosensing, structural elucidation via X-ray crystallography and entry into Phase I/II clinical trials. (2) Prospective (2026–): As depicted in the outer orange sector, the future focuses on establishing DNAzymes as next-generation nucleic acid therapeutics. Key frontiers include the development of smart DNAzyme materials, precision therapy, and deep-level innovations in intelligent design paradigms, delivery systems, safety assessments and industrialization paths. Some images are reprinted with permission from Ref. [66]. Copyright 1990, American Association for the Advancement of Science, Ref. [1]. Copyright 1994, Elsevier Ltd., Ref. [8]. Copyright 1995, Springer Nature, Ref. [7]. Copyright 1997, National Academy of Sciences, Ref. [252]. Copyright 2000, American Chemical Society, Ref. [253]. Copyright 2002, American Chemical Society, Ref. [254]. Copyright 2017, The Authors, and Ref. [40]. Copyright 2022, Springer Nature.

As an emerging modality in the nucleic acid therapeutics landscape, DNAzymes are now at a pivotal inflection point where the field must transition from foundational discovery to genuine clinical translation. Looking back, we have witnessed an acceleration of innovation driven by an end-to-end strategy spanning efficient selection, molecular engineering and biomedical contextualization. Looking forward, for DNAzymes to truly mature into next-generation nucleic acid therapeutics and molecular tools, profound advances will be required in design paradigms, delivery systems, safety and developability assessment and industrialization pathways (Figure 9).

First, intelligent and biomimetic design is an inevitable direction for DNAzyme engineering. Conventional SELEX is time-consuming and fundamentally constrained by library size, and future efforts must embrace data-driven design frameworks [240]. By integrating deep learning and artificial intelligence to decode high-dimensional relationships among sequence, structure, and function, the field is poised to shift from blind selection toward rational design [241]. Moreover, given the complexity of in vivo microenvironments, there is a pressing need to construct adaptive DNAzymes endowed with logic-gated control such that catalytic activity is unleashed only upon disease-relevant molecular cues [242]. In parallel, establishing in vivo SELEX and in vivo, like evolutionary platforms should facilitate the identification of intrinsically superior sequences with enhanced serum stability and improved cellular uptake [243].

Second, overcoming the delivery bottleneck will be decisive for clinical success [54,244]. Although technologies such as lipid nanoparticles and GalNAc conjugation have transformed the siRNA field, DNAzymes impose distinct constraints on delivery vehicles because their function is often conformation-dependent [245,246]. The field must move beyond passive reliance on the EPR effect in solid tumors and instead develop next-generation active targeting strategies, while benchmarking delivery efficiency and safety against clinically approved LNP formulations [247,248]. Key challenges ahead include evading reticuloendothelial system clearance, improving the efficiency of endosomal/lysosomal escape, and enabling synergistic integration with cell-based therapies or viral vector platforms.

Third, safety and druggability assessment urgently require standardization. As DNAzyme research increasingly advances into in vivo settings, immunogenicity and off-target effects can no longer be treated as secondary considerations. Beyond therapeutic efficacy, systematic evaluation is needed for risks associated with introducing exogenous DNA, including potential innate immune activation and gene-network remodeling under long-term administration [249,250]. Establishing standardized toxicology and pharmacokinetic evaluation frameworks tailored to DNAzymes, potentially adapted from the mature regulatory guidelines for ASOs and siRNAs, will be foundational for regulatory acceptance and eventual approval.

Finally, clinical positioning and industrial translation must be pursued through differentiated strategies. In a competitive landscape dominated by CRISPR, antisense oligonucleotides, and siRNAs, DNAzymes should seek first-in-class opportunities by leveraging their distinctive attributes, such as metal-ion dependence and efficient cleavage of specific RNA structural motifs, to address targets that remain difficult for other modalities [40,251]. From an industrial perspective, low-cost, scalable chemical synthesis constitutes a core competitive advantage. Importantly, DNAzymes should not be viewed as standalone interventions; rather, they are well-positioned to serve as powerful components within combination therapeutic regimens.

In summary, the next 5–10 years are likely to represent a golden window for the DNAzyme field. We anticipate closer collaboration through cross-disciplinary consortia involving chemists, immunologists, and clinicians to build a seamless pipeline from sequence design to clinical benefit. With continued innovation, DNAzymes may evolve into precise, controllable and broadly accessible molecular scalpels, opening new avenues for gene silencing and fine-tuned regulation.

## Figures and Tables

**Table 1:** Comparison of DNAzymes with other major nucleic acid therapeutic modalities.

Dimension	DNAzymes	ASOs	siRNAs	CRISPR-Based (Editing)
Core mechanism	Intrinsically catalytic RNA cleavage; activity often cofactor/structure dependent [16,17]	Mostly stoichiometric binding; effects via steric block or RNase H-mediated cleavage [18,19]	Functionally catalytic through RISC-mediated cleavage; guide strand reused [20]	Programmable nuclease editing; potentially durable after transient exposure [21]
Typical dosing implication	Potentially lower molar requirement if high turnover achieved; in vivo turnover often limited, so dosing advantage is context-dependent [16,17]	Often requires repeated dosing to maintain occupancy; durability depends on chemistry/tissue [18]	Potent; can show durable knockdown with intermittent dosing [22]	Potentially one-and-done effect (editing), but depends on delivery efficiency and safety constraints [23]
Off-target risk profile	Sequence-dependent mismatches; cleavage-site constraints may improve specificity; risks from partial complementarity and unintended RNA structures [16,17]	Hybridization-driven off-target binding; protein interactions (chemistry-dependent) [18]	Seed-mediated off-target repression; innate immune stimulation depends on chemistry/delivery [20]	Off-target genomic edits and on-target large rearrangements; guide-dependent [24]
Manufacturing complexity	Short, fully synthetic oligos; scalable solid-phase synthesis; catalytic core constraints may limit modification patterns [25]	Mature large-scale oligo synthesis with diverse chemistries; well-established CMC [18]	Mature oligo synthesis; duplex formation and formulation considerations [22]	More complex (gRNA and nuclease mRNA/protein; sometimes viral vectors); higher CMC burden [21]
Delivery constraints	Needs cellular uptake, correct folding and metal-ion microenvironment; stability and endosomal escape remain key [16]	Best established for liver/CNS; broad delivery toolbox [26]	Strongest in liver (LNP/GalNAc); extrahepatic delivery still challenging [27]	Payload size large; delivery often limiting; tissue targeting and immunogenicity critical [21]
Clinical maturity	Limited clinical experience; mostly preclinical, few candidates explored clinically (e.g., inhaled GATA-3 DNAzyme reported) [28]	High: multiple approved drugs across indications [19]	High: multiple approved drugs [22]	Emerging/early-to-mid: landmark approvals/late-stage trials exist, but modality still maturing [23]
Distinct best-fit niches	Targets benefiting from catalysis and modular retargeting, local delivery, or structured RNA motifs; also as activatable/logic-gated systems [16]	Knockdown/splice modulation where durable binding and chemistry optimization shine [19]	Potent knockdown where RISC pathway and delivery are favorable [22]	Genetic diseases where permanent correction is desired and safety/delivery acceptable [21]

**Table 2:** Summary of the selection of representative DNAzymes.

Catalytic Type	DNAzyme Name	Reaction Type	Typical Applications	References
Cleavage	10-23	RNA phosphoester cleavage	gene silencing/ biosensing	1997 [7]
Cleavage	8-17	RNA phosphoester cleavage	gene silencing/ biosensing	1997 [7]
Ligation	E47	Backbone ligation via formation of a 3′-5′ phosphodiester linkage	nucleic acid detection based on ligation reactions	1994 [1]
Ligation	CLDz2	Formation of a non-natural 1,2,3-triazole backbone linkage	Engineered as a ligation module for DNA nanostructure assembly, enabling applications in disease diagnostics and biosensing	2025 [3]
Modification	8JB210/7MN217……	N-acylation of nucleobases	investigate epitranscriptomic modifications/ site-specific oligonucleotide modification	2024 [69]
Modification	8LB203/11JP201……	Reductive amination-mediated N-alkylation	Site-specific covalent modification/labeling of nucleic acids for nucleic acid probe construction.	2024 [4]

## Data Availability

No new data were created or analyzed in this study. Data sharing is not applicable is not applicable.

## Abbreviation

The following abbreviations are used in this manuscript:

Abbreviation	Full term
AP site	apurinic/apyrimidinic site
ASO(s)	antisense oligonucleotide(s)
AuNP(s)	gold nanoparticle(s)
Cas9	CRISPR-associated protein 9
CHA	catalytic hairpin assembly
CLDz2	click-ligation deoxyribozyme 2 (DNAzyme-catalyzed triazole ligation)
COPD	chronic obstructive pulmonary disease
CRISPR	clustered regularly interspaced short palindromic repeats
CT	computed tomography
DMSO	dimethyl sulfoxide
Dz-seq (DZ-seq)	deoxyribozyme sequencing (high-throughput activity profiling by sequencing)
EPR	enhanced permeability and retention (effect)
FAM	6-carboxyfluorescein (fluorophore label)
GalNAc	N-acetylgalactosamine
G4	G-quadruplex
GATA-3	GATA binding protein 3
GPX4	glutathione peroxidase 4
HCR	hybridization chain reaction
HRV	human rhinovirus
HT-SELEX	high-throughput systematic evolution of ligands by exponential enrichment
LAMP	loop-mediated isothermal amplification
LFA	lateral flow assay
LNA	locked nucleic acid
lncRNA(s)	long non-coding RNA(s)
m1A	N1-methyladenosine
m^6^A	N6-methyladenosine
mRNA	messenger RNA
miRNA	microRNA
MOE	2′-O-methoxyethyl
MOF(s)	metal–organic framework(s)
MRI	magnetic resonance imaging
MsPA	mesyl phosphoramidate
NF-κB	nuclear factor kappa B
NIR	near-infrared
NLRP3	NLR family pyrin domain containing 3
PAGE	polyacrylamide gel electrophoresis
PCR	polymerase chain reaction
PD-L1	programmed death-ligand 1
PEG	polyethylene glycol
PET	positron emission tomography
PK-PD	pharmacokinetics/pharmacodynamics
PS	phosphorothioate
PDT	photodynamic therapy
RIP3	receptor-interacting protein kinase 3
RNA	ribonucleic acid
SB010	GATA-3 mRNA-targeting therapeutic DNAzyme candidate
SELEX	systematic evolution of ligands by exponential enrichment
SNP(s)	single-nucleotide polymorphism(s)
SuFEx	sulfur(VI) fluoride exchange
Th2	type 2 helper T
TNA	threose nucleic acid
TNF-α	tumor necrosis factor alpha
VEGF	vascular endothelial growth factor
VEGFR2	vascular endothelial growth factor receptor 2
XNA	xeno nucleic acid
